# Thyroglobulin Measurement Through Fine-Needle Aspiration for Optimizing Neck Node Dissection in Papillary Thyroid Cancer

**DOI:** 10.1245/s10434-021-10549-2

**Published:** 2021-08-12

**Authors:** Xi Jia, Yuanbo Wang, Yan Liu, Xiang Wang, Xiaobao Yao, Runyi Tao, Hui Liu, Aimin Yang, Rui Gao

**Affiliations:** 1 Department of Nuclear Medicine, The First Affiliated Hospital of Xian Jiaotong University, Xi’an, Shaanxi China; 2 Department of Ultrasound, The First Affiliated Hospital of Xi’an Jiaotong University, Xi’an, Shaanxi China; 3 Department of Otorhinolaryngology – Head and Neck Surgery, The First Affiliated Hospital of Xi’an Jiaotong University, Xi’an, Shaanxi China; 4 Department of Thoracic Surgery, The First Affiliated Hospital of Xi’an Jiaotong University, Xi’an, Shaanxi China; 5 Biobank, The First Affiliated Hospital of Xi’an Jiaotong University, Xi’an, Shaanxi China

## Abstract

**Background:**

Thyroglobulin measurement in fine-needle aspiration (FNA-Tg) is an additional diagnostic tool of lymph node metastasis (LNM) in papillary thyroid carcinoma (PTC). However, its performance as a preoperative indicator of lateral neck LNM in PTC is unclear. We evaluated the use of FNA cytology and FNA-Tg to detect neck LNM presurgery using a simple methodology, and established a cut-off value for diagnosing LNM in PTC.

**Methods:**

We performed a retrospective cohort study based on hospital records, including 299 FNA-Tg measurements from 228 patients with PTC. The cut-off value for FNA-Tg was obtained through a receiver operating characteristic (ROC) curve analysis. The relationships between various parameters and FNA-Tg were analyzed using Spearman’s correlation.

**Results:**

Of 299 lymph nodes (LNs) from 228 patients following surgery, 151 were malignant and 148 were benign. The median FNA-Tg levels were 414.40 ng/mL and 6.36 ng/mL in the metastatic and benign LNs, respectively. An FNA-Tg cut-off value of 28.3 ng/mL had the best diagnostic performance (93.38% sensitivity, 70.27% specificity, area under the ROC curve [AUC] 0.868) in the whole cohort. The diagnostic value performed better in the lateral neck group (level II–V, *n* = 163) than in the central neck group (level VI, *n* = 136); in the lateral neck group, the sensitivity and specificity of the FNA-Tg cut-off (16.8 ng/mL) were 96.25% and 96.36%, respectively.

**Conclusions:**

FNA-Tg is a useful technique for the diagnosis of LNM before surgery, especially in lateral neck dissection.

**Clinical trial registration number:**

ChiCTR1900028547.

**Supplementary Information:**

The online version contains supplementary material available at 10.1245/s10434-021-10549-2.

Thyroid carcinoma is the most common endocrine malignancy. Papillary thyroid carcinoma (PTC), which is frequently associated with cervical lymph node metastasis (LNM), accounts for most thyroid carcinoma cases.^1^,^2^ The extent of the initial surgical approach relies on the accurate diagnosis of regional nodal metastases, especially lateral LNM (LLNM).^3^–^5^ Although extensive dissection could achieve a better diagnosis for LLNM, it has a higher potential for nerve injury and hemorrhage. Therefore, lateral compartment dissection is only recommended with fair evidence of LLNM, as per American Thyroid Association (ATA) guidelines.^6^

Less-invasive approaches, such as ultrasound (US)-guided fine-needle aspiration cytology (FNAC), are therefore commonly used. Unfortunately, false-negative (6–18%) and non-diagnostic (up to 20%) results are common, resulting in high rates of misdiagnosis.^7^–^10^ To improve the diagnostic accuracy of FNAC, thyroglobulin measurement through FNA (FNA-Tg) is frequently practiced in the preoperative diagnosis of LNM in PTC. In most studies, FNA-Tg provides additional diagnostic value for locoregional recurrence post-surgery, although its cut-off values are still controversial.^11^ To avoid missing metastatic disease in patients who may benefit from lymphadenectomy, a combination of FNAC and FNA-Tg has been applied to accurately differentiate LLNM from benign LNs;^7^ however, it is unclear whether FNA-Tg measurements could effectively indicate lateral neck LNM in PTC.

In the present study, we aimed to investigate the diagnostic value of FNA-Tg for the detection of LNM from PTC presurgery using a simple methodology, especially the lateral lymph nodes (LNs), and thus guide the necessity of lateral neck dissection.

## Materials and Methods

### Patients

**Fig. 1:**
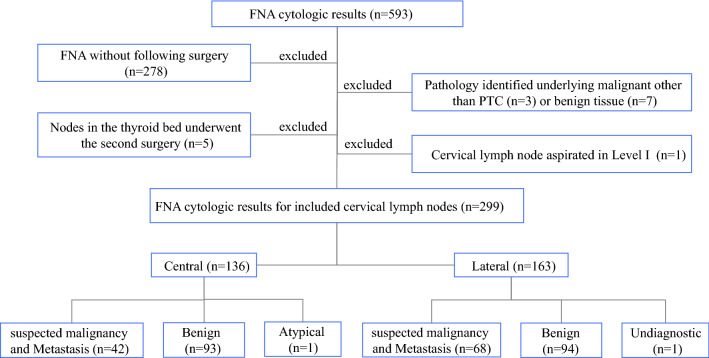
Study selection and standard reference. *FNA* fine needle aspiration, *PTC* papillary thyroid carcinoma

A total of 593 US-guided FNAs for cervical LNs were performed at The First Affiliated Hospital of Xi’an Jiaotong University in April–December 2019. A consecutive series of 445 patients with single or multiple suspicious cervical LNs underwent US-guided FNA to measure Tg levels (FNA-Tg). When multiple suspicious LNs were presented in one compartment, the representative nodes were selected for FNA. Only patients in whom PTC was confirmed by surgery were included in the study. Finally, a total of 299 LN samples from 228 patients were investigated (Fig. 1).

We categorized dissected LNs as central (level VI) or lateral (level II–V) based on the American Joint Committee on Cancer classification (Eighth Edition). In total, 136 central LNs and 163 lateral LNs were included. The study was approved by the Hospital Ethics Committee of the First Hospital of Xi’an Jiaotong University (XJTU-ZD10) and registered in the Chinese Clinical Trial Register (registration number ChiCTR1900028547).

### Ultrasound and Ultrasound-Guided Fine-Needle Aspiration

All 445 patients underwent US using a 10–12 MHz linear transducer prior to surgery, by experienced radiologists. Suspicious US characteristics included hyperechogenicity, cystic changes, calcification, abnormal vascularity, heterogeneous echogenicity, a round shape (longitudinal/transverse diameter ratio < 1.5), and a loss of echogenic hilum. The locations (levels II–VI) of all cervical LNs were recorded based on the guidelines of the American Joint Committee on Cancer classification.^7^,^12^ When discordant results existed among different examiners, agreement was reached after a joint review and discussion of the data.

US-guided FNA was simultaneously performed using 22-gauge needles. Every suspicious LN was aspirated twice to guarantee enough samples for analysis. The needle was repeatedly inserted into the LN until sufficient material filled the needle hub. Immediately after the first aspirate was expelled onto glass, 5 µL of the sample was added to 195 µL of Tg-free serum. The clear supernatant after centrifugation was used for subsequent FNA-Tg measurement, and the remainder of the aspirate was smeared for cytological examination (FNAC). After smearing the sample from the second aspiration, the remainder of the material was rinsed in saline for processing as a cell block. All smears were immediately placed in 95% alcohol for Papanicolaou staining. We classified the cytologic specimens into three categories—malignant, benign, and inadequate. If the cytology showed hemosiderin-laden macrophages, the specimen was classified as inadequate.

### Thyroglobulin Assessment

Tg was assayed using a monoclonal antibody immunoradiometric assay (IRMA; CIS Bios International, Gif-sur-Yvette, France). The functional sensitivity was 0.9 mg/L, analytical sensitivity was 0.2 ng/mL, and the interassay coefficient of variation (CV) was 6.9%. Values exceeding the maximum Tg were recorded as > 500 ng/mL. Serum Tg autoantibodies were measured using the Siemens IMMULITE 2000 (New York, NY, USA) assay (functional sensitivity, 40 kIU/L; analytical sensitivity, 20 kIU/L; and CV, 9%).

### Surgical Protocol and Histopathological Analyses

The extent of surgery was based on the results of FNAC + FNA-Tg. (1) When FNAC revealed malignant cells in the LNs, cervical neck dissection was performed along with total thyroidectomy. Cervical neck dissection involved central with/without unilateral/bilateral lateral compartment (levels II–V) dissection.^13^,^14^ (2) When the FNA-Tg levels were higher than 100 ng/mL in cases with negative cytological findings, neck dissection was also suggested in initial surgery.^14^,^15^ (3) In patients having LNs with suspicious US features but no definite metastatic cells found in FNAC and a level of FNA-Tg lower than 100 ng/mL, frozen section histologic analysis was only performed in cases required for it.

Final positive diagnoses required histological confirmation of metastatic thyroid carcinoma or clinical evidence of metastasis at follow-up, including an increased LN size observed by sonography and/or persistently increasing serum Tg. The examination was undertaken at least twice yearly. Negative diagnoses were cases free of metastatic disease by the same criteria, based on a negative thyroid-stimulating hormone (TSH)-stimulated Tg measurement or negative follow-up sonography.^13^

### Statistical Analyses

Data following a normal distribution are expressed as mean ± standard deviation (SD), and non-normally distributed data are expressed as median (interquartile range). The Chi-square test was used to compare counting data between groups, and measurement data were evaluated using Student’s *t*-test or Mann–Whitney *U* test. A receiver operating characteristic (ROC) curve analysis was used to determine the cut-off value of FNA-Tg for diagnosis of malignant LNs. The area under the ROC curve (AUC) and the confidence interval (CI) were also determined. Spearman’s correlation coefficients were used to estimate relationships between FNA-Tg and other parameters, while binary logistic regression was used to estimate multiple correlations between FNA-Tg results and other parameters. Using the pROC package in R, AUC, sensitivity, specificity, and 95% CIs were calculated under the negative binomial distribution. ROC curves were derived using the ‘pROC’ package in R (version 3.6.1; http://www.R-project.org).^16^,^17^ Other statistical analyses were performed in SPSS (version 24.0; IBM Corporation, Armonk, NY, USA). A *p*-value < 0.05 was considered significant.

## Results

### Patient Characteristics

The clinicopathologic characteristics of patients are summarized in Table 1. This study included 165 women (72.37%) and 63 men (27.63%), and patient ages ranged from 20 to 81 years (43.80 ± 11.85 years). Aspirated LNs were located at level II (*n* = 5), level III (*n* = 48), level IV (*n* = 101), level V (*n* = 9), and level VI (*n* = 136). Of the 228 patients who received thyroidectomy with neck dissection, 160 were given thyroidectomy with central LN dissection (CLND) only, 58 were given unilateral lateral LN dissection (LLND), and 10 were given bilateral LLND. A total of 265 (88.63%) LNs were ipsilateral and 34 (11.37%) were contralateral to the primary tumor. Metastatic LNs with bilateral cancers were considered ipsilateral LNs (27 patients with 36 LNs). Serum TSH and serum Tg levels did not differ between central LNs and lateral LNs (*p* = 0.237 and *p* = 0.295, respectively). Serum TgAb was higher in central LNs than in lateral LNs (*p* = 0.001). It is worth noting that LN sizes in the central neck were significantly smaller than those in the lateral neck (median 6.00 mm vs. 9.00 mm, *p* = 0.000) (Table 2).

**Table 1:** Patient characteristics

	Final outcome		LND outcome	
Total patients Total lymph nodes	228 299		158 205	
Age at diagnosis, years	43.80 ± 11.85	0.000^a^	41.67 ± 11.60	0.018^a^
Groups, < 55 years/≥ 55 years	185/43	0.010^b^	136/22	0.229^b^
Sex		0.009^b^		0.000^b^
Male	63 (27.63%)		119 (75.32%)	
Female	165 (72.37%)		39 (24.68%)	
Primary tumor				
BRAF	181 (79.39%)	0.372^b^	122 (77.22%)	0.884^b^
Multifocality	66 (28.95%)	0.170^b^	43 (27.22%)	0.027^a^
Hashimoto	60 (26.32%)	0.092^b^	56 (35.44%)	0.006^b^
Lymph node				
Location 1: Central/Lateral	136/163	0.289^b^	131/74	0.000^b^
Location 2: levels 2/3/4/5/6	5/48/101/9/136	0.670^c^	2/26/41/5/131	0.000^c^
Location 3: ipsilateral/contralateral	265/34	0.001^b^	198/7	0.238^b^

The follow-up results were only included in the final outcome, not in the LND outcome

*LND* lymph node dissection

^a^Derived from the two-sample *t* test

^b^Derived from the Chi-square test

^c^Derived from the Fisher’s exact test

**Table 2:** Clinical parameters comparation

	Central [*n* = 136]	Lateral [*n* = 163]	*p*-Value
FNA-Tg	156.75 (33.95, 500.00)	15.00 (0.00, 500.00)	0.000
Serum-Tg	9.71 (1.00, 19.25)	11.50 (1.31, 25.35)	0.295
TgAb	80.65 (18.48, 373.72)	38.10 (3.07, 121.90)	0.001
TSH	1.57 (0.98, 2.62)	1.42 (0.75, 2.35)	0.237
LNDmax	6.00 (4.00, 7.00)	9.00 (5.00, 13.00)	0.000

The follow-up results were only included in the final outcome, not in the LND outcome

*FNA-Tg* fine-needle aspirated thyroglobulin, *LNDmax* maximum diameter of lymph node, *LND* lymph node dissection, *TSH* thyroid-stimulating hormone, *Tg* thyroglobulin, *TgAb* thyroglobulin antibody

### Final Diagnosis

In the final diagnosis, 151 (50.50%) nodes were positive for metastatic disease and the remaining 148 (49.50%) were negative. The positive nodes were determined by histopathology (*n* = 130), cytology (*n* = 13), and follow-up evidence (*n* = 8). The 148 negative nodes were determined by histological diagnosis (*n* = 74) and follow-up sonography (*n* = 74).

FNA-Tg was higher in malignant LNs than in benign LNs in the central group, lateral group, and the total samples. The median FNA-Tg values for the lateral group measured in metastatic and benign LNs were 498.45 ng/mL (130.60, 500 ng/mL) and 0 ng/mL (0, 3.40 ng/mL), respectively. Other serum parameters revealed no significant differences between malignant and benign LNs in the lateral group (Table 3).

**Table 3:** Comparison of parameters according to the different groups

	Benign [*n* = 148]	Malignant [*n* = 151]	*p*-Value
*FNA-Tg*			
Whole	6.36 (0.00, 47.02)	414.40 (118.20, 500.00)	0.000
Central	49.40 (23.50, 257.40)	322.40 (91.20, 500.00)	0.000
Lateral	0.00 (0.00, 3.40)	498.45 (130.60, 500.00)	0.000
*Serum-Tg*			
Whole	8.47 (1.00, 19.90)	11.86 (1.32, 26.00)	0.106
Central	8.39 (1.00, 21.50)	10.05 (1.00, 18.70)	0.557
Lateral	8.47(1.00,19.10)	13.65 (2.51, 35.10)	0.077
*Serum-TgAb*			
Whole	69.40 (18.8, 206.60)	36.35 (2.67, 168.25)	0.035
Central	119.30 (27.80, 375.60)	54.40 (5.56, 373.10)	0.192
Lateral	51.45 (8.30, 131.92)	30.90 (1.00, 69.40)	0.061
*TPO*			
Whole	1.00 (1.00, 822.00)	1.00 (1.00, 592.00)	0.399
Central	124.40 (1.00, 1355.98)	1.00 (1.00, 543.00)	0.159
Lateral	1.00 (1.00, 374.60)	1.00 (1.00, 688.25)	0.920
*TSH*			
Whole	1.60 (0.88, 2.44)	1.37 (0.89, 2.43)	0.723
Central	1.68 (1.12, 2.49)	1.40 (0.89, 2.67)	0.903
Lateral	1.47 (0.75, 2.38)	1.36 (0.64, 2.35)	0.710
*LNDmax*			
Whole	7.00 (4.00, 11.00)	6.00 (3.75, 10.25)	0.345
Central	6.00 (4.25, 7.00)	5.00 (1.40, 7.00)	0.120
Lateral	9.00 (4.00, 13.00)	9.00 (6.00, 14.25)	0.629

*FNA-Tg* fine-needle aspirated thyroglobulin, *LNDmax* maximum diameter of lymph node, *LND* lymph node dissection, *TSH* thyroid-stimulating hormone, *Tg* thyroglobulin, *TgAb* thyroglobulin antibody, *TPO* Thyroid peroxidase

### Cut-Off Values for Thyroglobulin Measurement in Fine-Needle Aspiration (FNA-Tg)

**Fig. 2:**
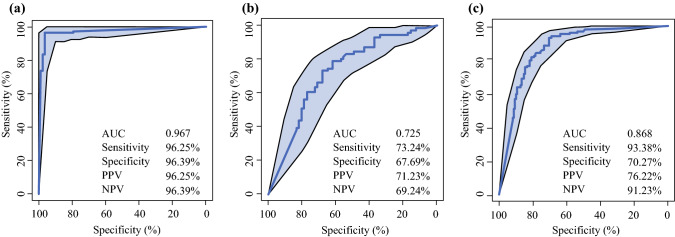
Receiver operating characteristic curve of the three groups. **a** Lateral group. **b** Central group. **c** Whole group. *PPV* positive predictive value, *NPV* negative predictive value, *AUC* area under the concentration-time curve.

In the lateral LNs (*n* = 163), an FNA-Tg cut-off of 16.80 ng/mL was the best diagnostic value, with 96.25% sensitivity and 96.39% specificity (AUC 0.967) (Fig. 2a). Negative and positive predictive values were 96.39% and 96.25%, respectively. An FNA-Tg cut-off of 119.85 ng/mL had the best diagnostic performance in the central cervical LNs (*n* = 136), with sensitivity and specificity of 73.24% and 67.69%, respectively (AUC = 0.725) (Fig. 2b). When taken as a whole, 28.3 ng/mL was the best cut-off value, with a sensitivity and specificity of 93.38% and 70.27%, respectively (AUC = 0.868) (Fig. 2c).

### Parameters Associated with FNA-Tg

To evaluate the factors associated with FNA-Tg levels in cervical LNs, we examined the correlations between FNA-Tg levels and other clinical parameters. Interestingly, only the serum TgAb levels for central LNs correlated negatively with FNA-Tg levels (Spearman’s correlation coefficient = − 0.232, *p* = 0.028), although the significant correlation of these two parameters was not observed in the lateral or whole groups. Serum Tg, TSH, or Hashimoto disease were not significantly correlated with FNA-Tg levels in all three groups (Table 4).

**Table 4:** FNA-Tg correlation with the clinical test results

	SerumTg	TgAb	TPO	TSH	Hashimoto
*Whole group*					
Correlation coefficient	0.079	− 0.086	0.009	− 0.025	− 0.011
*p*-Value	0.209	0.246	0.917	0.715	0.870
*Central group*					
Correlation coefficient	0.109	− 0.232	− 0.088	− 0.081	0.113
*p*-Value	0.238	0.028	0.492	0.404	0.267
*Lateral group*					
Correlation coefficient	0.135	− 0.101	− 0.017	0.036	− 0.056
*p*-Value	0.121	0.330	0.885	0.706	0.541

*FNA-Tg* fine-needle aspirated thyroglobulin, *TSH* thyroid-stimulating hormone, *Tg* thyroglobulin, *TgAb* thyroglobulin antibody, *TPO* Thyroid peroxidase

### Extent of Neck Dissection Based on Fine-Needle Aspiration Cytology and FNA-Tg

Further analysis was performed to evaluate whether the FNA-Tg cut-off value for diagnosing LNM could optimize the extent of surgery. The results for total thyroidectomy with LN dissection (LND) are summarized in Table 5. Among 131 central neck LNs, 92 (70.23%) demonstrated negative FNAC before surgery; however, postsurgical histopathological analyses revealed metastatic disease in 29 of these cases (29/92, 31.52%).

**Table 5:** FNA-Tg diagnosis, cytology and histology with total thyroidectomy with LND are summarized

	FNAC−		FNAC+	
	Histology+	Histology−	Histology+	Histology−
*Central group*				
Tg+	16	21	33	0
Tg−	13	42	6	0
*Lateral group*				
Tg+	4	1^a^	55	0
Tg−	1	11	2	0

*FNA-Tg* fine-needle aspirated thyroglobulin, *Tg* thyroglobulin, *FNAC* fine-needle aspiration cytology, *LND* lymph node dissection

^a^The inadequate cytology whose histology was negative was confirmed metastasis by 131-I imaging

The diagnosis of FNA-Tg performed better in lateral neck LNs than in central neck LNs. In total, 74 LNs with clear results via FNAC were removed during lateral LND (LLND), 17 of which (22.97%) showed FNAC-negative presurgery. In 5 of 17 LNs (29.41%), metastatic disease was detected after LLND. The FNA-Tg estimates for four of five metastatic LNs exceeded the validated cut-off of 16.80 ng/mL for diagnosing metastasis (median 46.30 ng/mL, range 8.50–500.00 ng/mL). It is noteworthy that FNA-Tg measurements for all benign LNs were < 16.80 ng/mL (median 0.00 ng/mL, range 0.00–12.20 ng/mL).

## Discussion

Although FNAC is the cornerstone in the diagnosis of cervical LNs, the accuracy highly depends on the experience of the cytopathologist and the adequacy of the aspirates. Clinicians consider FNA-Tg as an important adjunct to FNAC to avoid missing patients with metastatic disease. Our results demonstrated the added value of FNA-Tg for guiding surgical decisions for patients with PTC, especially for determining the need for LLND. In total, 11 of 94 cases (11.83%) were judged as ‘benign’, and 1 of 1 case (100%) judged as ‘inadequate’ by FNAC received LLND based on elevated FNA-Tg in LLN, and, in these cases, metastatic disease was revealed by postsurgical pathology. In most studies, the combination of FNA-Tg and FNAC showed a more optimal diagnostic yield than either FNAC or FNA-Tg alone.^7^,^18^,^19^ In the present study, the AUC of FNAC + FNA-Tg was higher than that of FNA-Tg alone (0.969 vs. 0.967), or that of FNAC alone (0.969 vs. 0.931) in the lateral group, although with no significant differences (*p* = 0.867 and 0.063, respectively). This suggested that using FNA-Tg alone without FNAC might be a choice in lateral LNs as it can offer comparable results at a much lower cost.^7^ The excellent negative predictive value could reduce the frequency of recytological examination, and the superior sensitivity could avoid the risk of reoperative complications, the extent of resection, and the cost of reoperation after the initial surgery.

In our study, the diagnostic accuracy of the whole group is consistent with that reported in previous research.^20^–^24^ Compared with the absolute value, the difference or ratio between FNA-Tg and serum Tg showed no diagnostic advantage (data not shown). However, the sensitivity and specificity values for the central group were much lower than their estimates for the overall group. LNs in the central region are more easily affected by blood contamination by circulating Tg at the time of sampling, or Tg leakage into the LN by lymph vessel draining.^7^,^25^,^26^ In the present study, only serum TgAb levels correlated negatively with FNA-Tg in the central LN group. It is quite possible that underestimation of the FNA-Tg concentration occurs, to some degree, in the presence of circulating TgAb in the central LN region but not in the lateral region.^27^ Consistent with correlation results, the number of TgAb positive cases with false negative results in the central group was significantly higher than in the lateral group (*p* = 0.023) [electronic supplementary table]. This might explain why LNs near the thyroid gland (e.g. paratracheal, pretracheal, and prelaryngeal nodes) before thyroidectomy yield inaccurate FNA-Tg values, irrespective of node pathology.^28^–^30^

FNA-Tg results are informative for LNs when the smallest diameter is < 10 mm. FNA-Tg is particularly recommended in cases involving small, partially cystic LNs, as a significantly increased rate of non-diagnostic cytological results are indicated in these cases.^19^,^31^,^32^ Our study highlighted the efficacy of FNA-Tg for small LNMs in the lateral neck. It is noteworthy that all nine LLNMs, not larger than 5 mm, were successfully identified by FNA-Tg, supporting its routine use in preoperative planning to determine the extent of lateral neck dissection. Further large-scale and high-quality clinical trials are warranted to confirm its effectiveness.

There is wide debate regarding FNA-Tg cut-off values. The high variation among FNA procedures and sample preparation protocols contributes greatly to the different cutoff values used in different studies.^33^–^41^ It is not surprising that these differences result in substantial variation in the detection of cell density in specimens among studies, which could lead to the observation of a wide range of FNA-Tg levels in PTC LNM.^41^ In this study, we compensated for this variation in sample dilution by adopting a quantitative analytic method in which a fixed amount of the FNA aspirate (5 µL) was diluted in Tg-free serum. The diagnostic value and best cut-off value were comparable with those reported in previous studies.^20^,^33^ Our standardized parameters, including the matrix type and volume of the aspirated sample, offer a potential standard operating procedure for further research, thereby enabling comparisons among studies.

Several limitations of the present study should be considered. First, the number of patients recruited was relatively small, and, additionally, other types of pathology other than PTC were not included in this study. FNA-Tg levels could be undetectable in some types of thyroid cancers, such as poorly differentiated thyroid cancer,^42^,^43^ which corresponds to the amount and intensity of Tg expression parallel with differentiation of the tumors. Furthermore, the number of metastatic disease cases, which was confirmed by postoperative histology, was relatively small, therefore future large-scale studies with longer follow-up periods are needed to verify our results. Second, we could not perform a node-by-node analysis for all LNs; instead, a level-by-level analysis was performed. Additionally, the diagnosis of most benign lateral LNs was based on follow-up results. Despite these limitations, this study has important clinical implications. Our data confirmed that FNA-Tg is a reliable method for the recognition of metastatic disease in the lateral neck. Optimized lateral neck node dissection for patients with PTC might be performed based on an FNA-Tg cut-off of 16.8 ng/mL. It is important to note that a patient whose cytology was inadequate, with an elevated FNA-Tg (433.6 ng/mL) in the lateral compartment, underwent the removal of 21 LNs in the lateral neck and showed no metastatic lesions by postsurgery pathology. However, radioiodine-avid disease in the aspirated compartment was detected in the post-therapeutic whole-body scan. Accordingly, an FNA-Tg value of > 16.8 ng/mL in LLN might be a strong indicator for the existence of metastatic disease, even when it is not revealed by postsurgical analyses.

## Conclusion

Diagnostic accuracies and cut-off values for FNA-Tg measurements differ between central and lateral cervical LNs. Regarding the diagnostic accuracy of lateral LNs, our standardized procedure was proved to be promising and should be considered to guide lateral neck dissection.

## Supplementary Information

Below is the link to the electronic supplementary material.

Supplementary file 1 (DOCX 14 kb)
