# Supplementary material for: Thyroglobulin Measurement Through Fine-Needle Aspiration for Optimizing Neck Node Dissection in Papillary Thyroid Cancer
Source: Ann Surg Oncol. 2021 Aug 12;29(1):88–96. doi: 10.1245/s10434-021-10549-2 (PMC8677638; doi:10.1245/s10434-021-10549-2)
Supplement: Supplementary file 1 — (DOCX 14 kb) [file 10434_2021_10549_MOESM1_ESM.docx]

**Supplementary table. The potential parameters of bias in the false negative and false positive results.**

|  | False negative | | | False positive | | | | | |
| --- | --- | --- | --- | --- | --- | --- | --- | --- | --- |
|  | TgAb | | | Serum Tg | | | Hashimoto | | |
|  | + | - | p value | + | - | p value | + | - | p value |
| Lateral | 0 | 3 |  | 1 | 2 |  | 0 | 3 |  |
| central | 15 | 4 | 0.023 | 13 | 8 | 0.550 | 11 | 10 | 0.223 |

Serum Tg+: >1 ng/ml.
